# Physicochemical Characterization of Starch and Cellulose Nanofibers Extracted from *Colocasia esculenta* Cultivated in the Colombian Caribbean

**DOI:** 10.3390/polym17172354

**Published:** 2025-08-29

**Authors:** Sandra Milena Daza-Orsini, Carolina Medina-Jaramillo, Alex López-Córdoba

**Affiliations:** 1 Grupo de Investigación en Bioeconomía y Sostenibilidad Agroalimentaria, Escuela de Administración de Empresas Agropecuarias, Facultad Seccional Duitama, Universidad Pedagógica y Tecnológica de Colombia, Carrera 18 con Calle 22, Duitama 150461, Colombia; smdaza@uniguajira.edu.co (S.M.D.-O.); carolina.medina02@uptc.edu.co (C.M.-J.); 2 Facultad de Ingeniería, Programa de Ingeniería Industrial, Universidad de la Guajira, Kilómetro 5, Riohacha 440002, Colombia

**Keywords:** agro-industrial byproducts valorization, bio-based polymers, circular economy, nanocellulose, underutilized crops

## Abstract

This study explores the valorization of *Colocasia esculenta* roots (flesh and peels) as a source of biopolymers by isolating and characterizing starch and cellulose nanofibers. Fresh roots were sourced from the Colombian Caribbean, and a bromatological analysis was conducted to determine their composition. Starch was extracted from the flesh (yield: 16.2 ± 0.5%) and characterized by a low amylose content (14.6 ± 0.9%) and a gelatinization temperature of 77.6 ± 0.3 °C. Granules showed spherical and polyhedral shapes and smooth, fissure-free surfaces. The median granule size (D50 = 12.2 ± 0.18 µm) exceeded several values reported for *Colocasia esculenta* from other regions. Cellulose nanofibers were isolated from peel byproducts (yield: 10.0 ± 1.4%), displaying dense fibrillar networks with diameters of 15–25 nm and lengths around 80 nm. FTIR analysis confirmed the presence of characteristic functional groups in both materials. Thermogravimetric analysis showed thermal degradation peaks at 320 °C for starch and 330 °C for nanocellulose. These findings demonstrate that *Colocasia esculenta*, an underutilized crop in the Colombian Caribbean, represents a promising and sustainable raw material for the development of bio-based polymers with suitable physicochemical, structural, and thermal properties.

## 1. Introduction

Growing concerns over climate change and the environmental impact of petroleum-derived polymers have stimulated increasing interest in renewable biopolymers [1].

Biopolymers are macromolecules naturally produced by living organisms or derived from renewable resources, including proteins, polysaccharides, and nucleic acids [2]. They can be obtained through plant extraction, microbial synthesis, or fermentation processes [2,3]. Due to their biodegradability, biocompatibility, and non-toxicity, biopolymers represent an attractive alternative to synthetic polymers in diverse applications, ranging from food packaging and agriculture to drug delivery and biomedical engineering [2,3].

Starch and cellulose are among the most abundant natural polymers on Earth, both polysaccharides but differing markedly in their structures and properties [4]. Starch consists of semi-crystalline granules primarily composed of two polyglucans: amylose, a mostly linear polymer of glucose residues linked through α-(1,4)-bonds with a few α-(1,6)-branches, and amylopectin, the major component, which possesses a highly branched structure with shorter chains and numerous α-(1,6)-linkages [4,5]. Conversely, cellulose is a linear polysaccharide composed of β-(1,4)-linked glucose units, whose hydroxyl groups promote extensive intra- and inter-molecular hydrogen bonding [4]. This bonding results in a compact, partially crystalline structure that provides high aspect ratio, mechanical strength, and resistance to enzymatic degradation. Although cellulose is insoluble in water, it can absorb water and swell, particularly in the amorphous domains [6,7]. Cellulose has several applications, including the production of films, hydrogels, and composites, as well as serving as a reinforcing agent in biodegradable materials for food packaging, biomedical devices, automotive and aerospace components, energy storage systems, and environmental remediation [4,8]. In plant cell walls, cellulose nanofibers are embedded within lignin–hemicellulose matrices [4,8]. Therefore, recent research has focused on isolating nanocellulose, mainly as cellulose nanofibers, cellulose nanomaterials, and cellulose nanocrystals, through chemical (alkaline or acid hydrolysis) and mechanical (ultrafine grinding, high-pressure homogenization) treatments [4,8]. These processes allow tailoring of nanocellulose properties to meet specific performance requirements [4,8]. Besides plant-derived sources, bacterial cellulose represents another important form of nanocellulose with high purity, crystallinity, and aspect ratio, which has been widely investigated for advanced applications [9,10].

Both starch and nanocellulose have been extracted from a wide range of agricultural sources and their byproducts. Common sources of starch include maize, potato, cassava, and rice [11], while common sources of cellulose include wood, cotton, seeds, leaf fibers, and agricultural residues such as wheat and rice straw [8]. It has been reported that the physicochemical characteristics of starch and cellulose are strongly dependent on the botanical source, genotype, and even local ecological conditions. In starch, the amylose–amylopectin ratio, granule size, shape, and crystallinity are highly variable across botanical sources, and these differences are closely linked to its gelatinization, retrogradation, enzymatic digestibility, and functionality in food and non-food applications [4,5,12,13,14,15,16]. For example, cereal starches typically contain smaller polygonal granules with lower swelling capacity, while potato starch presents large oval granules with phosphate substitutions that enhance swelling and viscosity [12]. Likewise, the fine structure of amylopectin, particularly the distribution of unit chain lengths, critically affects gelatinization and retrogradation properties, while amylose content and its biosynthesis are influenced not only by species but also by environmental factors such as climate, soil composition, and fertilization practices [15,16].

In the case of cellulose, its properties are not uniform across sources but are strongly influenced by factors such as degree of polymerization, degree of crystallinity, and morphological structure [17,18,19]. These parameters directly affect hydrolytic accessibility, mechanical behavior, and potential applications [17,18].

Among the wide diversity of tropical crops, *Colocasia esculenta* (commonly known as taro or malanga) has emerged as a promising yet underutilized starchy root with potential for integrated valorization within circular bioeconomy frameworks [2]. The corms of *Colocasia esculenta* are particularly rich in starch, which can account for over 85% of the total dry matter [20,21]. Additionally, peels, comprising between 15 and 30% of the total fresh weight of the root, are generated during the processing of *Colocasia esculenta* into various added-value products, as well as during the consumption of the roots [22]. These peels exhibit a high carbohydrate content (~93.5%), with low moisture (4.40–5.08%), ash (0.90–1.09%), and crude fiber (0.81–0.85%) levels [23]. These characteristics highlight the peels as a carbohydrate-rich byproduct with potential for further valorization.

Globally, *Colocasia esculenta* is widely cultivated across tropical and subtropical regions, particularly in Southeast Asia (e.g., China, India, the Philippines) and Africa (e.g., Nigeria, Ghana, Cameroon), where it plays a critical role in food security, nutrition, and small-scale agroindustry [24]. In addition, *Colocasia esculenta* is present throughout the Caribbean islands, as well as in Central and South America and the United States, where it has been described as an invasive species [25]. In Colombia, the cultivation of *Colocasia esculenta* is primarily concentrated in Caribbean region departments such as La Guajira and Cesar, where malanga is integrated into traditional agricultural systems, particularly among rural and indigenous communities. However, its industrial valorization remains limited and is mostly restricted to local food markets.

Previous studies have extensively reported on the physicochemical properties and potential applications of starches obtained from *Colocasia esculenta* cultivated mainly in Asia and Africa [21,26,27]. However, to date, the potential of *Colocasia esculenta* cultivated under the distinctive agroecological conditions of the Colombian Caribbean, characterized by semi-arid climates, moderate soil salinity, and low organic matter, remains few explored. In addition, no comprehensive studies have addressed the extraction and characterization of nanocellulose from *Colocasia esculenta* peels.

Therefore, this study aimed to valorize both the flesh and peels of *Colocasia esculenta* cultivated in the Colombian Caribbean through the extraction and physicochemical characterization of starch and cellulose nanofibers, respectively.

The novelty of this work lies not only in reporting the first systematic study of starch and cellulose nanofibers from Colombian-grown *Colocasia esculenta*, but also in demonstrating that an underutilized crop and its byproducts can provide functional performances comparable to other widely used sources. Such findings are particularly relevant for advancing sustainable materials research, as they support diversification of raw material supply chains, promote regional valorization, and open opportunities for integrating local crops into global bioeconomy strategies.

## 2. Materials and Methods

### 2.1. Materials and Reagents

Fresh *Colocasia esculenta* roots were sourced from a local market in Dibulla, La Guajira (Colombia). All reagents were of analytical grade (p.a.) and used as received without further purification. Potassium hydroxide (KOH, ≥85%, Merck, Darmstadt, Germany) was used for alkaline pretreatments, sodium chlorite (NaClO_2_, ~80%, Sigma-Aldrich, St. Louis, MO, USA) for delignification, and sulfuric acid (H_2_SO_4_, 95–97%, Merck, Germany) for acid hydrolysis. Ethanol (≥96%, Merck, Darmstadt, Germany), dimethyl sulfoxide (DMSO, ≥99.5%, Sigma-Aldrich, St. Louis, MO, USA), and urea (≥99.5%, Sigma-Aldrich, St. Louis, MO, USA) were used in amylose assays, together with iodine (I_2_, ≥99.8%, Merck, Darmstadt, Germany) and potassium iodide (KI, ≥99%, Merck, Darmstadt, Germany).

### 2.2. Bromatological Analysis of Colocasia esculenta Roots

The proximal composition of the *Colocasia esculenta* roots was performed directly on the raw material. Moisture, ash, protein (N × 6.5), lipid and crude fiber content were determined according to the methods of the Association of Official Analytical Chemists (AOAC) [28]. The carbohydrate values of the samples were determined by difference.

### 2.3. Starch Isolation from Colocasia esculenta Flesh

The starch extraction procedure followed a previously optimized method described by Medina-Jaramillo [29]. The roots were first washed with water, disinfected with a 250-ppm sodium hypochlorite solution, and peeled before being processed in an electric grinder. A mixture consisting of 1 kg of ground root and 2 L of water (1:2 ratio, selected based on preliminary trials and previous reports for efficient recovery) was then prepared and stored under refrigeration for 24 h [29]. The resulting suspension was filtered through cheesecloth to remove fibrous material, and the liquid phase was subjected to sedimentation. After, the supernatant was removed, and the remaining starch sediment was dried at 50 °C until it reached a moisture level below 12%. The final moisture content was determined gravimetrically by oven-drying samples at 105 °C until constant weight.

The dried starch was then ground using an electric mill (Cuisinart^®^ DCG-20BKN, Stamford, CT, USA) and sieved through a Tyler series 120 mesh, corresponding to a particle size smaller than 1250 µm.

The starch yield was calculated as the percentage of dry weight (dehydrated at 50 °C) of isolated starch related to the fresh weight of the root.

### 2.4. Extraction of Cellulose Nanofibers from Colocasia esculenta Peels

The procedure for isolating cellulose nanofibers from *Colocasia esculenta* peels was followed from the protocol established by Zuluaga et al. [30,31]. Peels (approximately 600 g per batch) were thoroughly washed, oven-dried at 100 °C for 24 h, then ground and sieved to a uniform particle size. The resulting material was then subjected to alkaline treatment by stirring in a 5 wt.% KOH solution for 14 h at room temperature.

The solid residue was delignified using a 1 wt.% sodium chlorite (NaClO_2_) solution at pH 5.0 and 70 °C for 1 h. This was followed by a second alkaline treatment with 5 wt.% KOH and a subsequent acid hydrolysis step using 1 wt.% H_2_SO_4_ for 2 h. After each stage, the remaining solid was thoroughly rinsed with distilled water until a neutral pH was reached. Finally, the purified cellulose fibers were subjected to mechanical fibrillation using an ultrafine friction grinder (Masuko Sangyo, Supermasscolloider, Satitama, Saitama pref, Japan), with the disc gap adjusted to −1. The final aqueous suspensions contained about 2% of cellulose nanofiber by weight. The yield of cellulose nanofibers was calculated as a percentage of the initial weight of *Colocasia esculenta* peels.

### 2.5. Characterization of Starch and Cellulose Nanofibers

#### 2.5.1. Amylose Content and Gelatinization Temperature of Starch Granules

The amylose content of *Colocasia esculenta* starch was determined by the iodine binding method [32]. A known mass of starch (~75 mg) was mixed with 10 mL of a urea-DMSO solution (1:9 *v/v*) and heated at 100 °C for 1 h. After cooling to room temperature, 0.5 mL of the solution were mixed with 5 mL of ethanol and centrifuged at 5000 rpm for 30 min. The supernatant was discarded, and the pellet was resuspended in 1 mL of urea-DMSO, 1 mL of I_2_/KI solution, and distilled water to a final volume of 50 mL. Absorbance was measured at 635 nm, and the amylose content was calculated using the following equation:

$$Amylose\;content\;\left(\%\right)=28.414\;\text{×}\frac{A_{635}}{2\;\times \;m_{starch}\times \;m_{solution}}\;\times \;100$$

where *m*_starch_ is the amount of starch used in the assay, *m*_solution_ is the weight of the solution, and *A*_635_ is the absorbance measured at 635 nm.

Gelatinization temperature of starch granules was analyzed using a Differential Scanning Calorimetry (DSC) instrument (Mettler Toledo, Greifensee, Schwerzenbach). A starch aqueous suspension was prepared in aluminum crucibles, sealed and kept at room temperature for 1 h before analysis. Measurements were performed from 40 °C to 90 °C at a constant heating rate of 10 °C/min under nitrogen flow (50 mL/min).

#### 2.5.2. Morphological Analysis and Particle Size

The starch granules were analyzed using a scanning electron microscope (JSM-6490LV, Jeol, Tokyo, Japan) operated at an accelerating voltage of 1.0 kV, beam current of 50 pA, and a working distance of 2.8 mm. Samples were mounted on aluminum stubs using double-sided adhesive tape and coated with a thin layer of platinum prior to imaging. For the analysis of cellulose nanofibers, the suspension was diluted 1:1000 in distilled water, sonicated for 15 min at 750 W, 20 kHz, and 20% amplitude, and subsequently stained with uranyl acetate. The prepared samples were observed using a field emission scanning electron microscope (FE-SEM) (Apreo™ 2, Thermo Fisher Scientific, Waltham, MA, USA) provided of STEM detector and operated at 30 kV, with a beam current of 13 pA and a working distance of 10 mm. The fiber diameter and length were calculated from the FE-SEM images using ImageJ software (version 2.0, GPL, National Institutes of Health, Bethesda, MD, USA).

Size distributions of starch granules were determined using a Mastersizer 3000 Laser Diffraction Particle Size Analyzer (Malvern Panalytical, Malvern, UK). For this purpose, starches were dispersed in ethanol (96% *v/v*) and diluted until an absorbance between 0.12 and 0.20 was obtained. The characteristic diameters D10, D50, and D90, representing the 10th, 50th (median), and 90th percentiles of the cumulative particle size distribution, were determined.

#### 2.5.3. Structural Analysis by Fourier Transform Infrared Spectroscopy (FTIR)

FTIR spectra of both starch and cellulose nanofibers were obtained using a Cary 660 FTIR spectrometer (Agilent, Santa Clara, CA, USA) equipped with an attenuated total reflectance (ATR) module. A total of 64 scans were recorded for each sample in the range of 4000–600 cm^−1^, at a resolution of 4 cm^−1^.

#### 2.5.4. Thermogravimetric Analysis (TGA)

TGA was conducted using a TGA/SDTA 851E thermogravimetric analyzer (Mettler Toledo, Zurich, Switzerland). Approximately 9.0 mg of each sample was placed in aluminum crucibles and heated from 30 °C to 800 °C at a rate of 10 °C/min under a nitrogen atmosphere. The curves that correspond to the first derivative of the TG curve (DTG) and the percentages of mass loss were obtained using the OriginPro Lab Sotfware version 9.0 64 bit.

## 3. Results and Discussions

### 3.1. Proximal Composition of Colocasia esculenta Roots

The results of the proximal analysis of *Colocasia esculenta* roots are showed in Table 1. These results are consistent with the general ranges reported in the literature but also reveal significant variation compared to *Colocasia esculenta* cultivated in other agroecological regions. For instance, Ferdaus et al. reported the proximate compositions of various *Colocasia esculenta* cultivars, showing a wide range of values for moisture (63–85%), protein (1.1–6.4%), fat (0.15–0.47%), crude fiber (0.6–3.6%), ash (0.6–4.8%), and carbohydrates (13–29%) [33]. Rojas-Sandoval et al. reported higher moisture content (70.64%) and slightly higher protein levels (1.5%) in raw *Colocasia esculenta*, along with lower fat content (0.2%) and higher crude fiber (4.1%). They also reported higher concentrations of calcium (43 mg/100 g) and phosphorus (84 mg/100 g), but lower contents of iron (0.55 mg/100 g) and sodium (11 mg/100 g) [34]. These variations in mineral composition among regions may be attributed to differences in soil nutrient availability, environmental conditions, and cultivar-specific uptake efficiencies.

Furthermore, Martins et al. reported the following proximal composition for raw purple *Colocasia esculenta* roots cultivated in Brazil: moisture 71.66 ± 1.13%, carbohydrates 21.61 ± 0.01%, protein 2.78 ± 0.09%, lipid 0.14 ± 0.02%, ash 2.43 ± 0.06%, and crude fibre 1.38 ± 0.03% [35]. Compared to roots from the Colombian Caribbean region, the purple cultivar showed higher moisture and protein contents but lower levels of carbohydrates and lipids. The ash content was nearly twice that found in the Colombian samples, while the carbohydrate content in Caribbean roots exceeded that of the purple variety. These compositional variations underscore the influence of genotype, agroecological conditions, and harvest maturity on the nutritional profile of *Colocasia esculenta* roots [33].

According to Mallilli et al. [36], when compared with other roots and tubers, *Colocasia esculenta* showed relatively low protein levels (≈1.1 g/100 g), comparable to cassava (≈2.4 g/100 g), potato (≈10 g/100 g), and sweet potato (≈3.4 g/100 g). Its fat content was also low (≈0.4 g/100 g), consistent with values typically observed in cassava, sweet potato, purple yam, and lesser yam. In terms of carbohydrates, *Colocasia esculenta* ranked among the richest sources within tropical roots, comparable to cassava and higher than potato and sweet potato. Regarding dietary fiber, the same authors reported that *Colocasia esculenta* corms contain 13.5 g/100 g, exceeding the values observed in cassava (4.6 g/100 g), potato (7.6 g/100 g), and sweet potato (8.1 g/100 g). Moreover, *Colocasia esculenta* fiber consists of both soluble (≈3.6 g/100 g) and insoluble (≈9.5 g/100 g) fractions, contributing to its nutritional value and potential prebiotic benefits.

### 3.2. Amylose Content and Gelatinization Temperature of C. esculenta Starch

The amylose content of the isolated starch from *Colocasia esculenta* was 14.6% ± 0.9, which aligns with previously reported values by Srichuwong et al. (16.3%) [37] and Rincón-Aguirre et al. (15.3%) [38]. These levels are notably lower than those found in common starches such as maize, wheat, or rice, which generally contain amylose contents ranging from 25% to 30% [39]. It has been reported that starches with low amylose content, like those from *Colocasia esculenta*, tend to form clearer and more soft and stable pastes, and exhibit reduced retrogradation [40].

**Figure 1:**
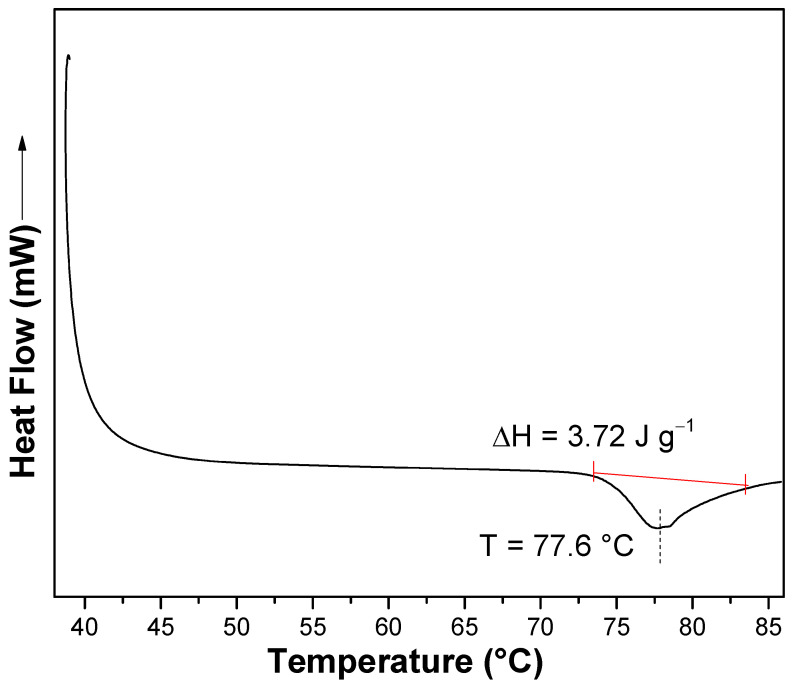
DSC thermogram of *Colocasia esculenta* starch.

The DSC thermogram showed a characteristic endothermic peak at 77.6 ± 0.3 °C (∆H = 3.72 ± 0.25 J/g), corresponding to the gelatinization of *Colocasia esculenta* starch granules (Figure 1). This result agrees with previous reports by Rincon-Aguirre et al., who reported a similar gelatinization temperature of 77.31 °C in *Colocasia esculenta* starch with a comparable amylose content [38]. In contrast, Lopes Dias et al. finds a lower gelatinization temperature of 72.7 °C in *Colocasia esculenta* starch samples containing a higher amylose content (23.31%) [41].

### 3.3. Yield and Morphology of Starch Granules and Cellulose Nanofibers

The starch yield from *Colocasia esculenta* was 16.2 ± 0.5%, consistent with the range reported for aroid crops (7–18.6%) using conventional methods such as wet extraction and centrifugation [42,43]. Variations in yield are primarily influenced by botanical origin, genotype, variety, harvest stage, granule size, and isolation technique [33].

**Figure 2:**
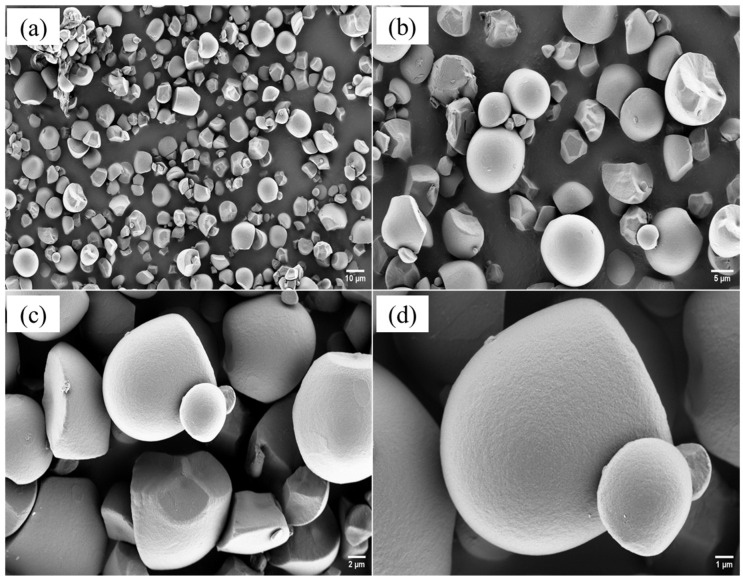
SEM micrographs of *Colocasia esculenta* starch granules at different magnifications: (**a**) 2000×, (**b**) 5000×, (**c**) 10,000×, and (**d**) 20,000×.

SEM images showed starch granules with diverse morphologies, including spherical, oval, and irregular polyhedral shapes (Figure 2). This morphology is consistent with previous descriptions of *Colocasia esculenta* starch, which typically report granules of spherical, oval, or polygonal form [26,33,44]. Moreover, the granules showed smooth surfaces without evident fissures or pores (Figure 2c,d). This contrasts with the findings of Mweta et al. for cassava starch granules, which, although generally smooth, often showed surface irregularities and fissures, reflecting greater heterogeneity in their morphology [26].

**Figure 3:**
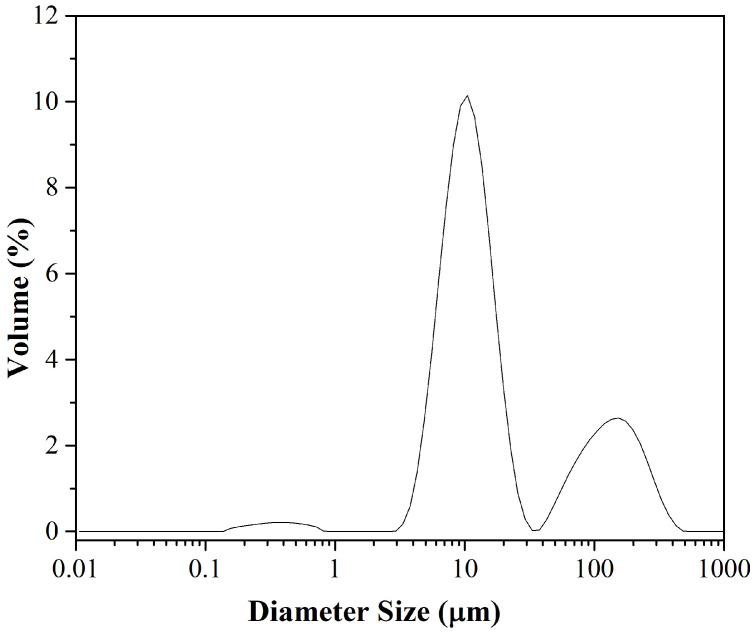
Particle size distribution of *Colocasia esculenta* starch granules.

Laser diffraction analysis showed the polydisperse nature of the *Colocasia esculenta* starch granules (Figure 3). The particle size percentiles values were D10 = 6.13 ± 0.14 μm, indicating that 10% of the granules had a diameter smaller than 6.13 μm; D50 = 12.2 ± 0.18 μm, corresponding to the median; and D90 = 149.0 ± 6.65 μm, showing that 90% of the granules had a diameter smaller than 149.0 μm. The granules size obtained in this study were larger than those reported by Rincón-Aguirre et al. (0.6–6 μm) [38], Lopes Dias et al. (mean size < 5 μm) [41], Martins et al. (3.66 μm) [35] and Mweta et al. (9.4–10.4 μm) [26], possibly due to differences in cultivar, agroecological conditions. They were also larger than those reported for cassava (11.4–12.9 μm) and maize (11.8 μm) [26]. In contrast, Koshenaj et al. observed larger granule sizes in lentil (D50 = 208.48 μm) and pea starches (D50 = 171.03 μm), while banana peel starch showed D50 values around 116.94 μm [45].

The observed variations in starch granule size and morphology compared to values reported in the literature can also be attributed to the combined influence of genotype, and agroecological conditions [15,16]. For instance, semi-arid climates and soils with moderate salinity, such as those of the Colombian Caribbean, may impose physiological adaptations that affect amyloplast development and the deposition of starch polymers [15,16]. Similarly, differences in nutrient availability and cultivation practices can modulate granule growth and shape [15,16].

The yield of cellulose nanofibers extracted from *Colocasia esculenta* peels was around 10 ± 1.4%, which is comparatively lower than the yields reported for *Colocasia esculenta* roots by other authors (15–65%) [46], as well as for various lignocellulosic agricultural residues such as banana peels (27–43%) [47], corncob bagasse and waste wood (25–34%) [48].

**Figure 4:**
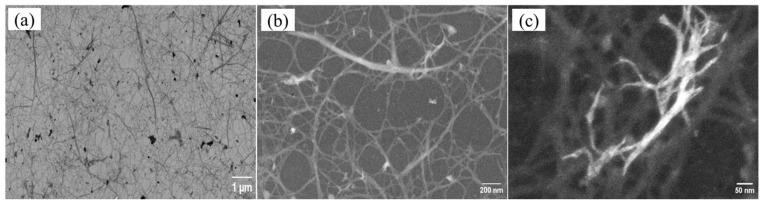
FE-SEM images of cellulose nanofibers extracted from *Colocasia esculenta* peels at different magnifications: (**a**) 25,000×, (**b**) 150,000×, and (**c**) 200,000×.

FE-SEM analysis showed an entangled fibrous network composed of nanofibrils with diameters between 15 and 25 nm and about 80 nm in length (Figure 4). Similar morphological features have been reported for cellulose nanofibers extracted from various agricultural residues such as banana peels and cassava root bagasse and peelings [47,49]. The observed diameters were comparable to those of nanofibers derived from banana peel bran (10.9–22.6 nm) [50], rice straw (12–35 nm) and potato tuber (15–55 nm) [51]. However, they were larger than those obtained from banana peel (2.89–4.65 nm) [47], lime residue (3–10 nm) [52] or coconut coir (5.6 nm) [53], likely due to differences in source material structure, processing methods, and fiber aggregation behavior.

### 3.4. Structural Features Revealed by FTIR

**Figure 5:**
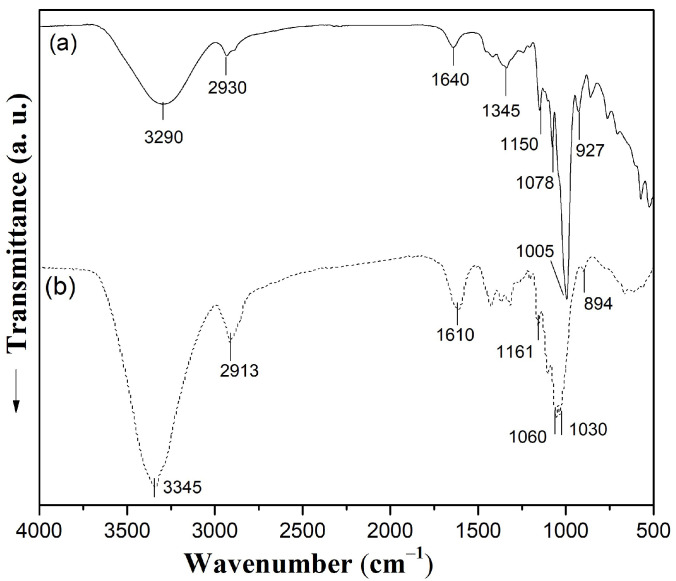
FTIR spectra of starch (**a**) and cellulose nanofibers (**b**) extracted from *Colocasia esculenta* roots.

The infrared spectra of starch and cellulose nanofibers extracted from *Colocasia esculenta* are presented in Figure 5. In the case of starch (Figure 5a), the FTIR spectrum showed characteristic absorption bands consistent with those reported in previous studies for *Colocasia esculenta* starch, as well as cassava and potato starches [29,38,54]. A broad band at 3290 cm^−1^ was attributed to the O–H stretching vibrations. The band at 2930 cm^−1^ was assigned to C–H stretching vibrations of –CH and –CH_2_ groups within the glucose units. The band at 1640 cm^−1^ was attributed to bending vibrations of adsorbed water molecules trapped within the starch matrix. Further distinctive bands were observed at 1345 cm^−1^ and 1150 cm^−1^, which can be associated with C–H bending and C–O and C–C stretching vibrations, respectively. The band at 1078 cm^−1^ could be caused by the C(1)-H bending mode of the starch glycosidic ring, while the signal at 1005 cm^−1^ was attributed to the C–O stretching in the anhydroglucose ring. The band at 930 cm^−1^ was attributed to α-1,4 glycosidic linkages in starches.

The FTIR spectrum of the cellulose nanofibers (Figure 5b) shows the characteristics signals of cellulose as reported in previous works [55,56]. A broad absorption band at 3345 cm^−1^ corresponds to O–H stretching vibrations associated with the hydroxyl groups involved in extensive intra- and intermolecular hydrogen bonding. The band at 2913 cm^−1^ was attributed to C–H stretching vibrations of aliphatic –CH_2_ groups. A peak at 1610 cm^−1^ was associated with the bending vibrations of adsorbed water molecules. Further, the peak at 1161 cm^−1^ was attributed to the asymmetric stretching of the C–O–C bridge in the β-1,4-glycosidic linkage of cellulose. The band at 1060 cm^−1^ was associated with the C–O and C–C stretching vibrations within the cellulose backbone, while the peak at 1030 cm^−1^ was attributed to the stretching vibrations of C–OH groups from primary and secondary alcohols. The absorption at 894 cm^−1^ is characteristic of the β-glycosidic linkages in cellulose and indicates strong hydrogen bonding between hydroxyl groups and ether oxygens located at the C3 and C5 positions of the anhydroglucose units. These features are consistent with cellulose isolated from *Colocasia esculenta* and other sources. For instance, Dominic et al. reported similar bands for cellulose nanofibers from *Colocasia esculenta* stems, noting the disappearance of the lignin-associated peak at ~1735 cm^−1^ after pretreatments, together with the persistence of cellulose-specific signals at 1030 and 892 cm^−1^, as evidence of effective lignin removal [20]. In the present study, lignin-associated peaks were not detected, also suggesting efficient elimination of non-cellulosic fractions during the isolation process.

Likewise, Leite et al. reported similar signals for cellulose nanofibers from cassava bagasse, particularly the O–H stretching at 3200–3500 cm^−1^ and the β-1,4-glycosidic band at 1162 cm^−1^ [49]. Tibolla et al. found comparable bands in banana peel-derived cellulose nanofibers, where the O–H peak at ~3337 cm^−1^ and the band at ~888 cm^−1^ confirmed cellulose structure after bleaching and enzymatic treatments [57]. Thai et al. also reported cellulose from sugarcane bagasse with absorption bands at 3331 cm^−1^, 2895 cm^−1^, 1160 cm^−1^, and 897 cm^−1^, which match the spectrum observed in this study [56].

### 3.5. Thermogravimetric Analysis of Starch and Nanocellulose

**Figure 6:**
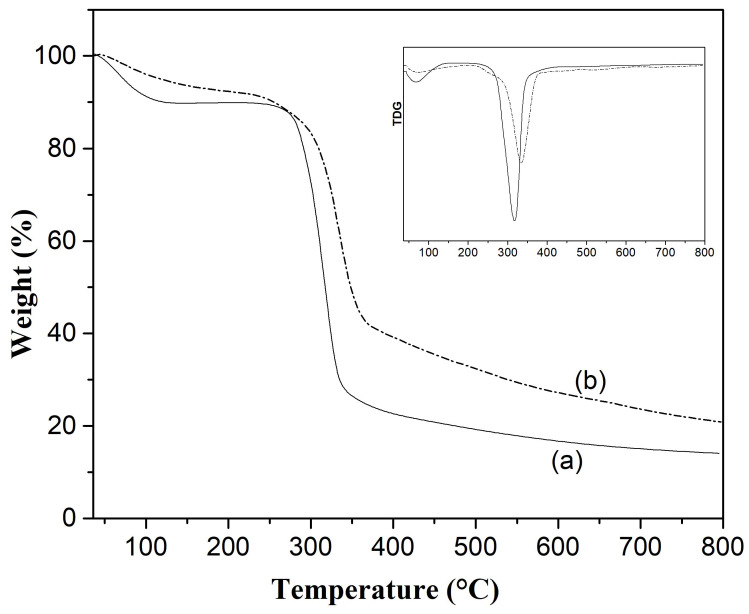
TG/DTG curves of starch (**a**) and cellulose nanofibers (**b**) from *Colocasia esculenta* roots.

Figure 6 shows the TGA curves of starch and cellulose nanofibers extracted from *Colocasia esculenta*. For starch (Figure 6a), the TGA profile showed two main weight loss events. The first, occurring below 150 °C (approximately 10% weight loss), was attributed to the evaporation of physically adsorbed water [44]. The second and most significant weight loss step (~65%) occurred between 280 °C and 340 °C, with a maximum degradation temperature near 320 °C, indicating breakdown of starch’s polysaccharide chains, including the cleavage of glycosidic linkages and volatilization of degradation products [44]. These findings are comparable to those reported by Martins et al. who observed that purple *Colocasia esculenta* starch exhibited an initial moisture loss between 30.0 and 154.7 °C, followed by a thermal stability region, and a major degradation phase occurring between 251.9 and 398.3 °C [35]. The variations in onset and peak degradation temperatures between studies may reflect differences in starch origin, botanical variety, residual non-starch components, or differences in amylose and amylopectin structure and content.

In the case of cellulose nanofibers extracted from *Colocasia esculenta* peels (Figure 6b), the TGA curve exhibited a two-step degradation pattern. The first stage, with a mass loss of approximately 8% occurring below 150 °C, was attributed to the moisture loss. The main degradation stage, accounting for ~50% of the total mass loss, occurred between 285 °C and 370 °C, with a maximum degradation peak around 330 °C. This major weight loss corresponded to the thermal decomposition of both amorphous and crystalline domains of cellulose, including the cleavage of β-1,4-glycosidic linkages and depolymerization of cellulose chains. A relatively low residual mass after 600 °C (below 20%) was also observed. These findings are consistent with reports from Dominic et al., who observed a similar two-step degradation behavior for cellulose nanofibers isolated from *Colocasia esculenta* stems [20]. Their CNFs exhibited the major weight loss (~50%) around 336 °C, slightly higher than the values observed in the present study.

Comparable degradation behaviors have been reported for CNFs derived from other biomass sources. For instance, Merais et al. found that cellulose nanofibers from *Musa acuminata* and *M. balbisiana* pseudostems showed the main weight loss within the range of 263–365 °C [58]. Similarly, Wang et al. reported the main weight loss at 357 °C for CNFs derived from bagasse [55]. Widiarto et al. observed a two-stage degradation process in cassava peel cellulose nanofibers, with an initial moisture loss under 150 °C and major decomposition between 200 °C and 600 °C [59]. Taken together, the thermal behavior observed for cellulose nanofibers from *Colocasia esculenta* peel in this study aligns well with literature data and confirms that the moderate-to-high thermal stability.

These findings also underline the relevance of *Colocasia esculenta*-derived starch and cellulose nanofibers as renewable biopolymers that can serve as sustainable alternatives to petroleum-based polymers. Their morphological and thermal properties suggest potential use in biodegradable films and composites, contributing to the development of eco-friendly materials aligned with circular bioeconomy strategies.

## 4. Conclusions

This study demonstrated the potential of *Colocasia esculenta* roots cultivated in the Colombian Caribbean as renewable feedstocks for bio-based polymers. The results revealed both similarities and distinctive features compared to other botanical sources, underscoring the influence of genotype and agroecological conditions on biopolymer properties. In terms of proximate composition, *Colocasia esculenta* corms cultivated in the Colombian Caribbean showed relatively low protein and lipid contents, but a comparatively high carbohydrate fraction.

Starch extracted from the corm flesh showed a relatively low amylose content of 14.6 ± 0.9% and a gelatinization temperature of 77.6 ± 0.3 °C. Morphological analysis showed granules with diverse shapes (spherical, oval, irregular polyhedral) and smooth, fissure-free surfaces. The median granule size (D50 = 12.2 ± 0.18 µm) exceeded several values reported for *Colocasia esculenta* from other regions.

Cellulose nanofibers obtained from the peels showed diameters between 15 and 25 nm, comparable to those reported for agricultural residues such as banana peels and rice straw. The absence of lignin-associated signals in the FTIR spectra suggested the efficient elimination of non-cellulosic fractions during the isolation process, while thermogravimetric analysis indicated moderate-to-high thermal stability, with degradation peaks near 330 °C.

From an applied perspective, the structural and thermal features of these biopolymers highlight their potential to be integrated into foods and biodegradable packaging’s. In particular, cellulose nanofibers from *Colocasia esculenta* peels show promise as reinforcing agents in biodegradable films, and composites materials where enhanced mechanical strength and high thermal stability are required.

Overall, this work provides the first systematic characterization of starch and cellulose nanofibers from *Colocasia esculenta* cultivated under Colombian Caribbean conditions. Beyond confirming functional comparability with widely used botanical sources such as cereals and wood-derived biomass, the results emphasize the relevance of valorizing underutilized tropical crops and their byproducts, contributing to supply chain diversification, regional development, and the advancement of circular bioeconomy strategies.

## Figures and Tables

**Table 1:** Proximal composition of *Colocasia esculenta* roots.

Parameter	Value (±SD)
Moisture (%)	64.05 ± 0.21
Protein (%)	1.23 ± 0.05
Fat (%)	0.67 ± 0.02
Carbohydrates (%)	30.88 ± 0.14
Crude fiber (%)	1.66 ± 0.04
Ash (%)	1.34 ± 0.03
Phosphorus (mg)	58.9 ± 0.02
Calcium (mg)	23.6 ± 0.01
Iron (mg)	0.78 ± 0.02
Sodium (mg)	68.9 ± 0.02

## Data Availability

The raw data supporting the conclusions of this article will be made available by the authors on request.
